# Chlorin Activity Enhancers for Photodynamic Therapy

**DOI:** 10.3390/molecules30132810

**Published:** 2025-06-30

**Authors:** Maciej Michalak, Jakub Szymczyk, Aleksandra Pawska, Marcin Wysocki, Dominika Janiak, Daniel Ziental, Marcin Ptaszek, Emre Güzel, Lukasz Sobotta

**Affiliations:** 1 Chair and Department of Inorganic and Analytical Chemistry, Poznan University of Medical Sciences, Rokietnicka 3, 60-806 Poznan, Poland; maciej.michalak2@student.ump.edu.pl (M.M.); 89318@student.ump.edu.pl (J.S.); aleksandra.pawska@student.ump.edu.pl (A.P.); marcin.wysocki@student.ump.edu.pl (M.W.); dominika.janiak@student.ump.edu.pl (D.J.); dziental@ump.edu.pl (D.Z.); 2 Doctoral School, Poznan University of Medical Sciences, Bukowska 70, 60-812 Poznan, Poland; 3 Department of Chemistry, Biochemistry University of Maryland, Baltimore County (UMBC), 1000 Hilltop Circle, Baltimore, MD 21250, USA; mptaszek@umbc.edu; 4 Department of Engineering Fundamental Sciences, Sakarya University of Applied Sciences, 54050 Sakarya, Türkiye; eguzel@subu.edu.tr

**Keywords:** PDT, nanotechnology, chlorin e6, liposomes, PACT

## Abstract

Photodynamic therapy (PDT) is a non-invasive therapeutic method with over a century of medical use, especially in dermatology, ophthalmology, dentistry, and, notably, cancer treatment. With an increasing number of clinical trials, there is growing demand for innovation in PDT. Despite being a promising treatment for cancer and bacterial infections, PDT faces limitations such as poor water solubility of many photosensitizers (PS), limited light penetration, off-target accumulation, and tumor hypoxia. This review focuses on chlorins—well-established macrocyclic PSs known for their strong activity and clinical relevance. We discuss how nanotechnology addresses PDT’s limitations and enhances therapeutic outcomes. Nanocarriers like lipid-based (liposomes, micelles), polymer-based (cellulose, chitosan, silk fibroin, polyethyleneimine, PLGA), and carbon-based ones (graphene oxide, quantum dots, MOFs), and nanospheres are promising platforms that improve chlorin performance and reduce side effects. This review also explores their use in Antimicrobial Photodynamic Therapy (aPDT) against multidrug-resistant bacteria and in oncology. Recent in vivo studies demonstrate encouraging results in preclinical models using nanocarrier-enhanced chlorins, though clinical application remains limited.

## 1. Introduction

The non-invasive approach of photodynamic therapy (PDT) has been recognized for over a century. Nevertheless, it has yet to be fully utilized to its fullest potential. The idea of PDT originated in Munich at the beginning of the 20th century when Oscar Raab discovered the antimicrobial properties of acridine red, which are such that, when exposed to light, it becomes toxic to *Paramecium* spp. [1]. Soon after, von Tappeiner, together with Jodlbauer and dermatologist Jesionek, began researching fluorescent dyes for skin tumors. The approach was eventually named “photodynamic” by von Tappeiner in 1907 [2]. To this day, PDT has found various clinical applications in dermatology, ophthalmology, dentistry, and cancer treatment. With 104 currently recruiting clinical trials and 382 completed ones, the number of applications for this approach continues to expand [3]. This growing interest highlights the need for a deeper understanding of PDT’s underlying molecular mechanisms.

Three crucial elements constitute the molecular mechanism of effective PDT. The first is a photosensitizer (PS), the second is light of an appropriate wavelength that can penetrate the tissue and excite the PS, and the third is the presence of molecular oxygen [4]. When illuminated, PS molecules can undergo three different photochemical mechanisms [5,6]. Both Type I and Type II mechanisms begin similarly; when the PS in its ground singlet state, (^1^PS^0^) absorbs the energy in the form of photons. One of its electrons becomes excited. It has been established that, in the process of transitioning between molecular orbitals, an electron typically exits the most energetically favorable orbital in order to enter a higher energy orbital. This process is accompanied by the preservation of the electron’s spin orientation, leading to the formation of an excited singlet state within the PS molecule (^1^PS*). The ^1^PS* can only exist for a very short time, reaching few nanoseconds, because of energy loss through non-radiative (internal conversion) or radiative (fluorescence) decay. However, during this state, part of the excited ^1^PS* molecules can undergo intersystem crossing (ISC), leading to electron spin reorientation, yielding the excited triplet state (^3^PS*), which has a considerably longer lifetime (µs) [7]. Reorientation of the electron’s spin is induced by spin–orbit coupling (SOC), an internal magnetic interaction within the electronic shell of the PS molecule. The ground oxygen molecule is in the triplet state (^3^O_2_) and has two electrons with parallel spins located on two molecular orbitals, which makes the interaction between ground state ^3^O_2_ and ground state ^1^PS^0^ forbidden by the spin selection rule. Thus, the SOC and the stage of ISC ^3^PS* are crucial for the electronic interaction between the PS molecule and O_2_ [8]. During the Type I reaction, the ^3^PS* accepts an electron from a reducing agent, i.e., nicotinamide adenine dinucleotide NADH, forming a radical anion (PS^−•^), which can then transfer the electron to molecular oxygen, forming a superoxide radical anion (O_2_^−•^). O_2_^−•^ formation can be followed by further transformation to other reactive oxygen species (ROS), like hydrogen peroxide (H_2_O_2_) and hydroxyl radical (HO^•^). Then, the ROS can damage the biomolecules or the cell’s organelles, like the cell membrane, ribosomes, nucleic acids, etc., resulting in necrosis or apoptosis of targeted cells. As for the Type II mechanism, ^3^PS* reacts directly with environmental molecular oxygen in its ground state (^3^O_2_). This can result in physical energy transfer, yielding excited-state singlet oxygen ^1^O_2_* and a ground-state singlet PS molecule, ^1^PS^0^. ^1^O_2_* is considered to be a non-radical ROS with high oxidizing potential and electrophilic properties. Due to its high reactivity, ^1^O_2_* is characterized by a short aqueous environment lifetime (3.5 µs) and is considered to be responsible for cellular damage during PDT [5,6,8,9,10].

By contrast, the least prevalent Type III mechanism is hypothesized to be an anomaly on account of its oxygen independence, as opposed to the oxygen dependence of the other two types of mechanisms. An excited-triplet-state ^3^PS* can enter this pathway by reacting directly with residing nearby biomolecules like nucleic acids, proteins, or other macro-biomolecules. After forming a complex with a Type II PS, the biomolecule may be destroyed by PS in its excited state [5,6,11]. Psoralens are known to enter this mechanism; however, ongoing research aims to develop novel Type III PSs, as they can avoid limitations imposed by hypoxic conditions, which are common in cancer tissues or microbe-infected sites. Type III PSs may also demonstrate even greater selectivity toward specific types of biomacromolecules (e.g., psoralens-DNA, NBES-RNA) [5,11]. The most common mechanisms in PDT are Type I and Type II, with their prevalence depending largely on oxygen availability, the local environment, and the chemical nature of the PS. For instance, in the chlorin–albumin complexes, Type I prevails over Type II; however, the bacteriochlorin Zr-TBB obtained by Luo et al. demonstrated equally distributed generation of ROS and ^1^O_2_* [12,13].

**Figure 1:**
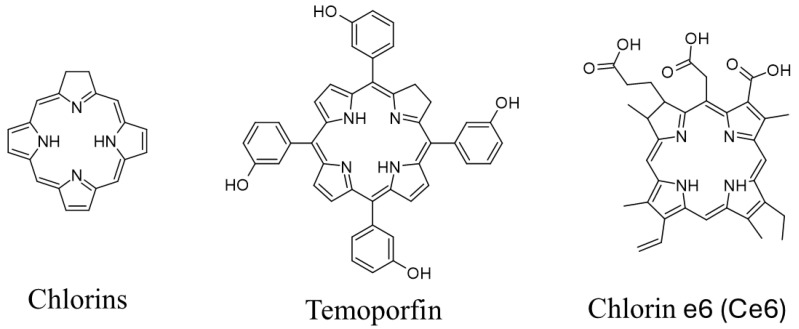
Structures of chlorins. These images were created with the use of ACD/ChemSketch (Freeware) 5 January 2024.

Chlorins (Figure 1) are a group of tetrapyrrolic macrocycles that resemble chlorophyll, a naturally occurring porphyrin derivative with a reduced peripheral cross-conjugated double bond [14,15,16]. They exhibit high absorbance in the red region of the visible spectrum (650–750 nm). They also exhibit high quantum yields of ^1^O_2_* generation (45–60%) and lower dark toxicity than porphyrins’ [15,17]. Chlorins have clinically approved representatives such as chlorin e6 (Ce6, Figure 1), which is used in the treatment of gliomas and lung cancer [18].

Temoporfin/Foscan^®^/Meso-tetra-hydroxyphenyl-chlorin (mTHPC, Figure 1) is another chlorin-based PS, used in the treatment of advanced head, neck, pancreatic, and prostate cancers. Temoporfin is one of the most potent PS utilized in PDT; its high activity can be associated with good tissue accumulation and solubility, granted by the meta-hydroxyls present on arylic moieties in meso positions. However, it presents some significant drawbacks, such as rapid photobleaching and photosensitizing of the skin that may last for several weeks [18].

Despite promising results of PDT against cancer or bacteria, it still struggles with some key factors to become fully successful. One of them is the bulkiness of the PS molecules, which are mostly represented by macrocyclic compounds with poor water solubility, and possibly limited availability [19]. Nanoparticles (NP) are designed to increase availability; however, they can also help to decrease the effect of hypoxic conditions of the treated tissue [20,21]. Owing to the addition of other active substances, a complex of NP and PS, is able to achieve improved effectiveness. For instance, the PDT used against certain types of cancer can induce latent up regulation in the TGF-*β*1 expression, causing PDT to turn out not effective. By creating programmed nanocomplexes to release the TGF-*β*1-inhibitors after the activation of the PS, the success rate of the therapy may rise significantly [22]. It is also possible to design NP to respond to the tumor microenvironment by releasing PS only in desired conditions of the target. When making an ROS-sensitive conjunction between PS and nanomolecules, the PS is inactive, until this conjunction breaks in the programmed conditions [23]. This approach not only addresses the availability obstacle but also decreases the activation of the PS outside of targeted tissue, thus reducing unwanted phototoxicity in off-target locations. Until now, it was possible to use various types of nanomolecules for the improvement of PDT. Although variety is impressive, they can be grouped as follows: lipid-based, polymer-based, carbon-based, bio-molecule-based, and non-organic-based. While these nanoparticle systems have primarily been explored in the context of cancer treatment, their adaptability also opens the door for applications beyond oncology. One particularly promising area is the fight against multidrug-resistant infections.

The growing problem of globally increasing bacterial antibiotic resistance encourages the search for alternatives more than ever [24]. The *Enterococcus* spp., *Staphylococcus aureus*, *Klebsiella pneumoniae*, *Acinetobacter baumanii*, *Pseudomonas aeruginosa*, and *Enterobacter* spp. (ESKAPE) bacteria especially pose a threat to public health, and are a major concern of WHO from 2017 [25,26]. The decades-long studies on PSs allowed for the development of Antimicrobial Photodynamic Therapy (aPDT), also known as Photodynamic Antimicrobial Chemotherapy (PACT). The method relies on the same rules as PDT. The oxidation stress has a destructive effect on microbes’ cellular structures, proteins and cell walls [27]. These events ultimately lead to the eradication of pathogens. In consideration of the mechanism of aPDT, it is possible to use it against various types of pathogens: Gram-negative bacteria, Gram-positive bacteria, fungi, viruses or protozoa [28,29,30,31]. Another advantage of aPDT is that, until today, there has not been any crucial resistance mechanisms discovered that could significantly interfere with the mechanism of action nor the therapeutic outcome [32]. However, there are some reports of possible tolerance rising due to sublethal doses of light and sensitizer [33]. Biofilms are complex architectural structures built of bacteria and the matrix of extracellular polymeric substances. The matrix consists of polysaccharides, DNA and proteins, which allows bacterial cells to stick to each other and strongly adhere to the surfaces [34]. Biofilms exhibit resistance to various eradication procedures, often causing contaminations or infections, which are challenging in treatment. With increased intrinsic resistance, it is more likely for bacteria in the biofilms to develop antibiotic resistance due to easier horizontal gene transfer and the aforementioned matrix environment [34]. The aPDT seems to be a suitable tool in coping with biofilms as, up until now, the data is promising. Compared to chlorhexidine (a common chemotherapeutic used in periodontal infections) in a multispecies biofilm, chlorin e6 (PS) exhibited much stronger eradicating potential [35,36]. Nanocarriers are more than suitable for improving the penetrability and efficiency of photodynamic eradication.

**Figure 2:**
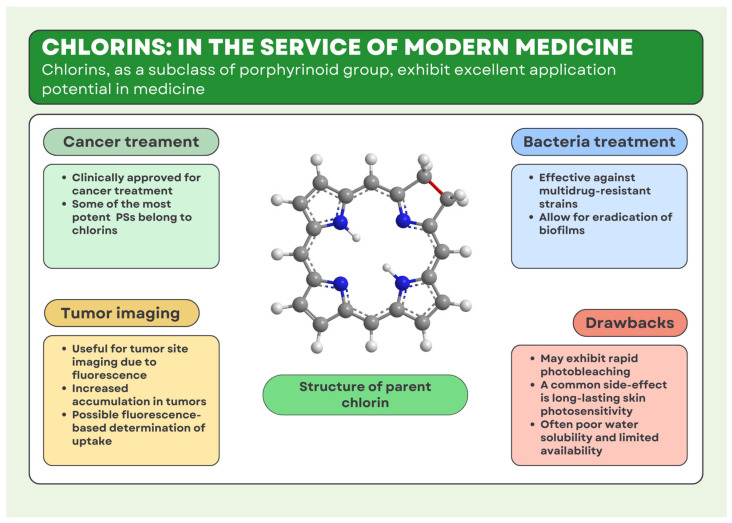
Applications and possibilities of chlorins in medicine. These images were created with the use of Avogadro Version 1.2.0 and Canva.com.

Alongside its promising PACT applications (Figure 2), PDT has also continued to evolve as a valuable strategy in oncology, providing a targeted and minimally invasive alternative to conventional cancer therapies. The use of PDT has proven to be an effective treatment for both benign and malignant tumors [37]. The most important aspect of this method is the ability of cancer cells to accumulate a PS in their structure, which is a source of ROS after irradiation. The method was first used to treat tumors in 1903. Forgotten for a while, decades ago, the therapy was rediscovered in the 1970s. Then, Ivan Diamond et al. [38] published their research conducted against cancers of the nervous system. They presented observations on the most popular of the porphyrinoid groups—the porphyrins. It has been demonstrated that malignant tumor cells exhibit a greater capacity for capturing PS molecules than healthy cells. The introduction of PS into glioma cancer, followed by exposure to radiation with a wavelength of 620 to 640 nm, resulted in the destruction of the tumor in the tested rats, with no negative effects on healthy cells. The therapy developed in this way offered a novel approach to tumors and cancers. In the following years, the possibility of using various PSs and others to treat tumors such as breast cancer or bladder cancer was confirmed [39]. The dense structure of the tumor makes it difficult for the PS to penetrate into the tumor and this effect is also exacerbated by the inability of the drug to reach tumor cells that are away from the blood vessels, ultimately leading to reduced exposure to PSs [40,41]. Another significant problem is that, after administration of a systemic PS, the drug tends to accumulate in the patient’s skin structures. It causes one of the most common adverse effects of the PDT, which is hypersensitivity to light and susceptibility to sunburn [42].

## 2. Lipid-Based Nanocarriers

**Figure 3:**
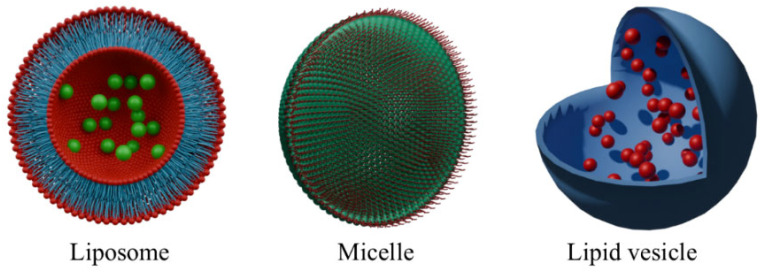
Representation of lipid-based nanoparticles. Approximation of average sizes of: liposome ~100 nm, micelle 10–100 nm, lipid vesicle 20 nm–50 μm. These images were created with the use of Blender 4.1.

Nano-lipids, like liposomes, micelles, or lipid vesicles (Figure 3), are widely used in PDT. These platforms are perfect to deliver poorly water-soluble PSs. Moreover, they are biocompatible and degradable (Table 1). By using them, it is possible to achieve controlled and prolonged release [43]. Liposomes are better than other nanoplatforms at targeting specific areas. Their shape, resembling a vesicle, allows for loading them with the PS, while the outer layer of the sphere can be decorated with targeting molecules, e.g., antibodies or nutrient molecules for tumors, like folic acid. Another advantage caused by the spherical shape of the liposomes is that they can be programmed to release encapsulated molecules in response to specific conditions, e.g., pH, temperature, enzymatic activity, magnetic, ultrasound, redox potential, and electric field [44].

### 2.1. Lipidic Vehicles

Liposomes are lipid-based drug carriers considered to be safe. Their properties ensure an enhancement in drug stability and an improvement in their pharmacokinetics, often allowing for good accumulation inside the tumor cells. Based on their structure of bilayer vesicles, the liposomal formulations provide various ways of PS encapsulation, either inside the vesicle itself, or inside the lipid bilayer. The site of encapsulation is strongly associated with the structure of PS, its substituents, properties, and affinity to both lipid bilayer and internal medium. If the PS is in its water-insoluble form (for example, Ce6), its affinity is higher toward the lipid bilayer rather than the aqueous internal medium. On the contrary, if PS is water-soluble (sodium salt of Ce6), its affinity toward the aqueous internal medium is higher than toward the lipid bilayer. Despite the site of encapsulation and different encapsulation efficiency, liposomes tend to enhance the activity of encapsulated PS [45,46]. Liposomes bearing two FDA-approved PSs, Ce6 and methylene blue (MB), were investigated toward human cervical normal and cancer cells. The combined-PSs liposomes showed low dark toxicity, significantly lower than liposomes bearing Ce6 or MB alone. When irradiated, Ce6-MB liposomes revealed higher decreases in both cancer and normal cell viability due to high singlet oxygen generation, although normal cells were affected to a lesser extent. Additionally, the encapsulated PSs promoted the apoptosis of tumor cells rather than necrosis. Compared to liposomes bearing single PS, the Ce6-MB liposomes showed superior activity toward cells upon irradiation [47]. The combination of PS with cytostatic is also a known and versatile strategy, allowing for dual action toward malignant cells. For example, Ce6 was co-encapsulated with paclitaxel (PTX) inside the pH- and photoresponsive liposomes made of lecithin and PEG conjugated with orthoester moieties and lutein. The orthoester moiety allowed for controlled release inside the tumor cells due to their lowered pH. In addition, the photoresponsive lutein allowed for controlled release upon light irradiation. The liposomes showed increased drug release under irradiation combined with a low-pH tumor environment. The activity of liposomes was assessed toward the 4T1 human breast cancer cell line, and the results indicated that combined Ce6 and PTX provided the highest decrease in cell viability, although the activity of free PTX was not much lower, in contrast to that of free Ce6 [48]. The strategy based on PS and a cytotoxic drug may be enhanced by increasing oxygen content inside the tumor cells. Liposomes may contain, for example, a compound that mimics the action of enzymes, such as catalase (CAT). One of the compounds that exhibit CAT-like action is manganese (IV) oxide. This phenomenon is associated with the ability of MnO_2_ to decompose hydrogen peroxide present in tumor cells into water and oxygen, increasing its levels available for PS to utilize. The liposomes containing Ce6, PTX and MnO_2_ were tested against HeLa cells in vitro and U14 (mouse cervical carcinoma) tumor in vivo. The combination of MnO_2_, PTX, and Ce6 exhibited the highest rate of cell killing and highest inhibition of tumor growth, indicating the synergistic action between MnO_2_-produced oxygen and the PS, additionally enhanced by the action of PTX [49]. In contrast to increasing the oxygen content, the strategy that assumes combining the PS with inhibitors of crucial life and proliferation processes, such as glycolysis, is also a versatile one. In such an approach, a PS (Ce6) may be combined with 3-bromopyruvate (3BP), a potent glycolysis and mitochondrial respiration inhibitor, able to inhibit the tumor cells’ energy supply and consequently their growth. Using energy blockers is considered a starvation therapy, as it is oriented toward inhibition of crucial metabolic pathways. This phenomenon may also enhance the activity of PS itself, as less oxygen is consumed by the tumor, and hence the tumor microenvironment (TME) remains less hypoxic. Additionally, the use of liposomes ensures sufficient cell internalization, confirmed by in vitro fluorescent imaging. The cellular uptake is crucial for the effectiveness of PDT and starvation therapy. Such combinations were tested against HeLa cells, xenografted on nude mice, as well as in vitro. The system, consisting of liposomes loaded with Ce6 and 3BP, revealed the highest effectiveness in reduction in cancer cell viability and tumor size and weight compared to Ce6 and 3BP alone [50]. Similarly, altering the TME can be achieved by combining PDT and coagulation-targeting liposomes. Ce6-loaded liposomes were utilized to either damage tumor vasculature or force the formation of blood clots, as well as to deepen the hypoxia inside the tumor. The formed blood clots were then targeted by another liposomes, with a coagulation-targeting A15 peptide, ensuring high selectivity of delivery. Those liposomes were also equipped with a specific drug activated by tumor hypoxia banoxantrone dihydrochloride (AQ4N), which additionally suppressed tumor growth. Such a cooperative approach may be a versatile strategy toward highly hypoxic tumors, as both types of liposomes may exhibit a synergistic action [51]. Another study that proves that liposomes can be successfully used with chlorins was carried out by Chen et al., who developed a liposome system for effective aPDT. The cationic, pH-sensitive liposomes loaded with ultra-small copper oxide (Ce6@Lipo/UCONs) were tested against MRSA biofilms [52]. The Minimal Inhibitory Concentration (MIC) was assessed in two pH values, 7.4 and 5.5, to research the controlled release of Ce6 and copper oxide. The MIC of Ce6@Lipo/UCONs in the pH of 7.4 was 512 µg/mL; however, it decreased significantly to 32 µg/mL in the pH of 5.5. Moreover, the MIC of Ce6@Lipo/UCONs in the pH of 5.5 decreased even further to 8 µg/mL when bacteria were irradiated with NIR (near-infrared light) with a power density of 100 mW/cm^2^ [52]. This data proves the occurrence of photodynamic effects and the controlled release from the liposomes. The ablation effect of the biofilm with the Ce6 irradiated or not was not present, while the Ce6@Lipo/UCONs when irradiated caused an 83% reduction in mass of the biofilm [52]. Comparing it to 29% of UCONs (ultra-small copper oxide) and 28% of Lipo/UCONs in both dark and irradiated conditions, the synergism between liposomes Ce6 and UCONs was proved [52].

### 2.2. Conjugates with Micelles

Micelles, due to analogous properties to liposomes, may be utilized in a similar manner. The encapsulation of PSs, such as Ce6 with cytostatic such as camptothecin (CPT) [53] PTX [54,55] or sorafenib (SRF) [56], is a popular approach, as the combination of PDT with traditional chemotherapy usually provides better results than both therapies alone. Such micelles can be built of cationic polypeptide conjugated with PSs, which can spontaneously assemble into micelles, simultaneously encapsulating the conventional drug. Additional modification with pH-vulnerable PEG moieties can either reduce the surface charge or improve the targeting properties of the micelles. Lowered-pH TME can then force the detachment of PEG chains, exposing the cationic micelle moiety and therefore increasing the binding between the micelle and tumor membranes. Then, PDT can be performed by either opening the inner micelle and releasing the drug or performing classic PDT action [53]. Similarly, the structure of the micelles can be obtained, for example, from linear or hyperbranched polymers modified with thioether or thioketal groups that, due to their hydrophobicity, exhibit good stability, but are also prone to oxidation by ROS. Oxidation of those groups into hydrophilic sulfoxide or sulfone groups leads to micelle opening, providing a controlled, ROS-responsive manner of releasing the drugs. While the amount of intratumoral ROS may not be sufficient for opening the micelles, the action of co-encapsulated PS can be enough to initiate a proper drug release without impairing the photodynamic activity. Such a manner provides synergistic action between PDT and classic cytostatic treatment [54,55,56]. As in liposomal control of TME, micellar formulations can also be designed to either increase oxygen level, or take advantage of hypoxia-activated drugs [57,58,59]. The oxygen level increase, in order to enhance the PDT efficacy, can be achieved by incorporating oxygen carriers, such as perfluorinated crown ethers (PFCEs), inside the micelle along with the PS. The ability of carrying oxygen by PFCE is provided by van der Waals interactions between fluoride moieties and oxygen. It allows for enclosing sufficient amounts of oxygen, therefore increasing its amount available for PDT [57]. Instead of carrying molecular oxygen, one can utilize its production in situ, with the use of drugs that possess oxygen-generating potential, such as a tetravalent platinum-based diazido complex. This octahedral complex undergoes photoreduction to Pt(II) complexes, also yielding radicals, nitrogen and oxygen, particularly in the presence of intratumoral H_2_O_2_. Simultaneously, the reduction of Pt(IV) to Pt(II) occurs in the presence of glutathione (GSH), leading to its consumption in the process, additionally decreasing the antioxidative regulation of the cell. This results in increased level of oxygen, ROS and singlet oxygen produced in combined Pt complex and Ce6 action [58]. On the contrary, the micelles can be designed to take advantage of the hypoxia in a combined PDT-AQ4N approach, based on the synergy of PDT, simultaneously blocking the energy supply chain of the tumor, also resulting in increased PDT efficiency due to lowered tumor oxygen consumption [59].

**Table 1:** Advantages and disadvantages of lipid-based NP.

Type of Nanoparticle	Advantages	Disadvantages
Liposomes	Effective encapsulation and improved pharmacokinetics of photosensitizers [45,46]. Ability to combine PSs with synergistic therapeutic agents (chemotherapeutics, oxygen generators) [49,51,52].	Limited physicochemical stability under physiological conditions [45,46]. Risk of non-specific biodistribution and premature drug leakage [49,51,52].
Micelles	Precise, stimuli-responsive drug release (ROS, pH) [53]. Synergistic combination of PS with chemotherapeutics or oxygen-generating agents [54,55,56].	Dependence on sufficient intratumoral ROS or external activation [58]. Stability and predictability challenges in complex biological environments [54,55,56].

## 3. Polymer-Based Nanocarriers

**Figure 4:**
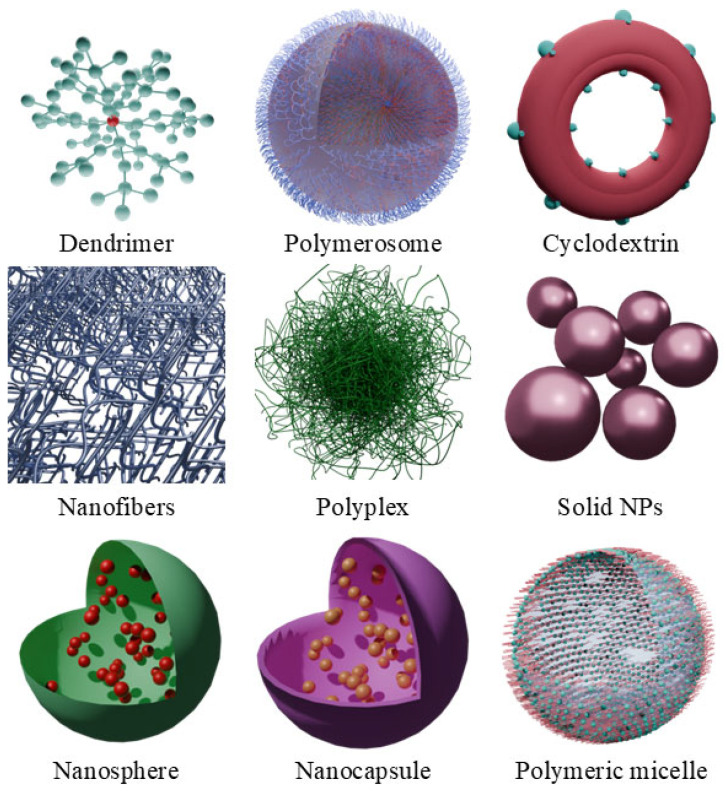
Representation of polymer-based nanoparticles. Approximation of average sizes of: D endrimers 1.1–9.8 nm, polymersomes 20 nm–600 μm, cyclodextrins 1.46–1.75 nm, nanofibers 10–500 nm, polyplexes 20–100 nm, solid nanoparticles 50 nm and 300 nm, nanospheres 180 nm, nanocapsules 200 nm, polymeric micelle 10–100 nm. These images were created with the use of Blender 4.1.

The most widely used group in PDT is polymer-based nanocarriers. Polymer-based complexes (PBCs) are characterized by numerous advantages: good PS binding properties, biodegradability, extended stability and biocompatibility (Table 2). Moreover, during the design and manufacturing process of PBCs, it is easier to control the characteristics of particles and keep them more size homogenous, compared to self-assembling structures, e.g., micelles [60]. Similarly, to lipid particles, PBCs’ surface can also be modified with additional molecules improving targeting or overlay enhancing therapeutic process. The wide choice of frameworks of the polymer nanocarrier is another advantage in designing therapeutically effective conjugations, as polymers can be manufactured in shapes like dendrimers, polymerosomes, nanospheres, nanofibers, nanocapsules, solid NP, polymeric micelles, polyplexes or cyclodextrins (Figure 4) [60]. The most important feature of a polymer for medical application is biodegradability, which is achieved by the use of materials like Poly(p-dioxanone) (PPDO), Chitosan, Dextran, Poly(hydroxybutyrate) (PHB), Poly(hydroxybutyrate-co-hydroxyvalerate) (PHBV), Gelatine, Poly(L-lysine), Polylactide, Poly(Lactide-co-Glycolide), Poly(caprolactone), Polyglycolide, Poly(butylene succinate) (PBS), Starch-polyvinyl alcohol, Starch-PLA, Polycarbonate, Polyurethanes, Cyclodextrins, Cellulose, Poly(β-amino ester), Alginate, and other polysaccharides from marine and plant sources [60]. To increase selectivity towards triple-negative breast cancer (TNBC), comb-like polystyrene particles were conjugated with two PSs (porphyrin and octaethylporphyrin). Additionally, nuclease-resistant RNA aptamers were added, creating complexes with high affinity towards characteristic TNBC surface receptors. The specific aptamers immensely increased the intracellular uptake of the complexes; moreover, the aptamers in the used concentrations remained biocompatible, exhibiting minimal cytotoxicity.

### 3.1. Chlorins and Cellulose

Cellulose is a natural polymer consisting of two anhydroglucose units joined by a *β*-glycosidic bond [61]. It has the desirable properties of a nanocarrier such as a developed and chemically modifiable surface area, high physical resistance, biocompatibility, degradability, and sustainability [61]. Sharma et al. used carboxymethyl cellulose (CMC), pectin (PC), and sodium alginate (SA) to develop biopolymeric films embedded with Chlorin p6 (Cp6) or MB. The PACT activity was tested against MRSA, and the biocompatibility of developed films was also tested with human keratinocyte (HeCaT) cells [62]. The hydrated films exhibited high transmittance of 65–70% in the range of 500–750 nm. After 1 h of incubation, they irradiated the Cp6(2µM])SA-PC-CMC film, with 660 nm wavelength light. The results were dependent on the dose of light and embedded PS. For 10 J/cm^2^, the photobactericidal effect was the highest and equal to a 3.5 log reduction in MRSA without causing dark toxicity [62]. In comparison, free Cp6 in 2µM concentration achieved a 4.5 log reduction in the bacterial cells. In contrast, the free MB achieved better effects than the embedded MB films [62]. The proposed approach of using natural and sustainable biopolymers to create films with PS can lead to the development of new types of dressings for infected and hard-to-heal wounds. In another study, the Ce6 loaded into the polydopamine-modified carboxymethyl cellulose hydrogel was utilized as a potential PS formulation. The CMC was amidated with dopamine moieties (CMC-DA), and subsequently oxidized with sodium periodane, simultaneously maintaining polymerization of the dopamine moieties. The polymerization resulted in the formation of the hydrogel, and the by-forming sodium iodide was encapsulated inside, forming a CMC-PDA-NaI (CDI) hydrogel. The CDI was then dried and soaked in an aqueous–ethanolic solution of Ce6. The obtained CDI/Ce6 hydrogel was then tested toward cancer maintained from SW1990 human pancreatic cancer cells, injected into the back of nude mice. The hydrogel could perform either PDT or PTT, and both approaches, as well as their combinations, were investigated. The separate PDT and PTT approaches significantly slowed down the tumor growth, with a slight advantage toward PTT, although the tumors themselves were not eliminated (ca. 100–150% growth after 28 days, compared to over 650% growth in control). On the other hand, the combined PDT/PTT approach resulted in the eradication of the tumor few days after the treatment, with no signs of recurrence or growth [63].

### 3.2. Chlorins and Chitosan

The chitosan is also an example of a naturally derived biopolymer. Yue et al. developed hydrophilic hydroxypropyl chitosan (HPCS) carriers for Ce6, forming HPCS-Ce6 conjugates with better bactericidal profiles against *S. aureus*, and *E. coli* [64]. They obtained three types of conjugates with different Ce6:HPCS mass ratios: HPCS-Ce6-1 = 5:100, HPCS-Ce6-2 = 10:100, HPCS-Ce6-3 = 20:100. When irradiated with 660 nm light, the Minimal Inhibitory Concentration MIC achieved by HPCS-Ce6 HPCS-Ce6-3 was 1.8 μg/mL, compared to free Ce6 31.2 μg/mL against S. aureus. Without irradiation, the HPCS-Ce6-2 and HPCS-Ce6-3 achieved Minimal Bactericidal Concentrations (MBCs) of 7.8 μg/mL, while the MBC of free Ce6 was determined as 31.2 μg/mL [64]. The PACT effectiveness of developed conjugates against *E. coli* was significantly lower. The MIC and MBC for all three HPCS-Ce6 were, respectively, 500 μg/mL, and 1000 μg/mL, and for the free Ce6, these were 250 μg/mL, and 500 μg/mL [64]. Moreover, the bacterial binding capacity studies showed that, although the HPCS-Ce6 interacts with the *E. coli* cells, the colony itself remains immune to the generated ROS. The cause may lie in the structural characteristic cell exterior of Gram-negative bacteria or the HPCS that, when conjugated, reduces the effectiveness of the free Ce6. The increase in water solubility positively affected the PACT effectiveness against *S. aureus*. These Gram-positive bacteria seemed to be considerably more prone to the ROS generated as a result of the photodynamic activation of Ce6 [64].

Chitosan-based particles, due to their plethora of free functional groups, are prone to modification. For example, the free amino groups of chitosan can be modified with the addition of inhibitors of ROS scavengers, such as phenethyl thiocyanate. Its incorporation can increase the accumulation of ROS, providing better PDT effects, particularly in terms of apoptosis induction and DNA damage. Interestingly, the phenethyl thiocyanate moieties provided good cytotoxicity even without light irradiation, showing an interesting behavior that can be utilized in the future [65]. The TME-responsible chitosan-based formulations were also investigated: the chitosan was modified with perfluoroheptanoic acid conjugated with Ce6, and used for incorporation of CAT with the utilization of its fluorophilicity. The system presented good membrane penetration due to fluorinated moieties, and owing to its pH responsiveness and oxygen-generating properties was able to enhance the PDT efficacy by decomposing the intratumoral hydrogen peroxide [66]. The ROS-responsive chitosan-based particles were tested as well. They contained the pinacol phenylborate linked to chitosan by thioketal moiety—both considered ROS-sensitive. Such a system was used to encapsulate Ce6, and due to its response to photodynamic effect and intratumoral ROS, provided controlled Ce6 release. The proposed chitosan and Ce6 combinations were tested for ROS generation in relation to free Ce6 and reduction in cell viability against YD-38 (gingival squamous cell carcinoma), KB (keratin positive oral epidermal carcinoma), and SCC-15 (tongue squamous cell carcinoma) lines. In both parameters, ROS generation and lowering cell viability, the combination of Ce6 and chitosan proved to be superior in comparison to free Ce6 [67].

### 3.3. Chlorins and Fibroin

Silk fibroin (SF) is a natural polymeric protein that can be obtained from cocoons of the silk worm *Bombyx mori* [68]. Besides its wide use in the textile industry, it has also found a place in medicine as an FDA-approved natural biomedical material. This fibrous protein is not only exceptionally biocompatible and weakly immunogenic, but also, due to its origin, it has good biodegradability and a low environmental impact [69]. Moreover, SF can be manufactured into a wide variety of nanoparticles, e.g., hydrogels, films, scaffolds, nanofibers or microspheres. SF comes in three different structural forms: Silk I, Silk II, and Silk III. Silk I mainly consists of *α*-helixes and coil structures [68]. Due to its structure, Silk I has a hydrophilic character; on the other hand, Silk II is poorly water-soluble. The scaffold of Silks II mainly consists of *β*-sheets, making it the most stable form. Silk III is the least common form, which is mainly present at the water–air interface [68]. Considering these properties, SF can successfully meet the challenges posed by different active excipients in various routes of administration and delivery approaches.

Li et al. took advantage of SF by fabricating electrospun films, which were aligned with Ce6 (SFCF@Film) [70]. The SFCF@Film was then tested in male BALB/c mice wound models. The wounds were infected with *S. aureus* suspension and then treated with SF film or SFCF@Film. After being irradiated with 660 nm wavelength, the wounds were observed for 10 days. The wounds treated with SFCF@Film exhibited antibacterial properties towards *S. aureus*. Moreover, the authors report that SFCF@Film created a cell-friendly scaffold that promoted M2 polarization of macrophages and accelerated the growth of fibroblasts, which ultimately resulted in the facilitated process of wound healing [70].

### 3.4. Chlorins and Polyethyleneimine

Polyethyleneimine (PEI) is a synthetic polymer that has a long history of use in the medical industry. Owing to its mechanical properties and flexibility, PEI is used in biomedical products such as implants, dental materials or drug-delivering particles [71]. A unique trait of PEIs is their high density of positive charges, and a dynamic branch-like structure that facilitates interactions with functionalized compounds [71]. Despite concerns about PEI’s toxicity and biocompatibility, these parameters can be improved by simple modifications, e.g., conjunctions with PEG, dextran or hyaluronate [71]. Due to its highly interactive nature, it is possible to improve the hydrophilic character of the PEI or develop a hydrophobic derivative [71]. Wang et al. used PEI as functionalizing agent with graphene to create a loadable nanocomposite (PEI-G). The PEI-G was then loaded with Ce6, forming (PEI-G@Ce6), and assessed for enhancement of PACT activity against *S. aureus* [72]. The PEI-G exhibited an antibacterial effect in high concentrations; moreover, the irradiated PEI-G@Ce6 exhibited a stronger antibacterial effect than the PEI-G alone. While the photostability of PEI-G@Ce6 increased compared to the free Ce6, the singlet oxygen generation was weakened. As ^1^O_2_ plays a major role in PACT activity, the decreased generation of it resulted in an MIC of 4 µg/mL and an MBC of 21 µg/mL, which are significantly higher compared to the reported MIC and MBC of amoxicillin (0.42 µg/mL and 0.56 µg/mL, respectively), a common antibiotic drug used against Gram-positive bacteria [72].

### 3.5. Chlorins and PLGA

The properties of PLGA and its biocompatibility often create opportunities to encapsulate various systems in order to achieve a more satisfactory delivery, and possibly an enhancement in their action. The systems compatible with PLGA may be based, for example, on graphene quantum dots (GQDs), constituting a carrier for other molecules, such as PSs and chemotherapeutic drugs. The GQDs were conjugated with PEG moieties terminated with amine groups through amide bonds and subsequently underwent another amidation with Ce6 and further conjugation with gamabufotalin moiety. The NPs were then coated with PLGA, and, in parallel, the NPs of PLGA-coated capsaicin (PLGA-Cap) were prepared. The Cap with targeting improved by PLGA coating was supposed to dilate tumor vasculature, leading to less hypoxic environment, and to improve the accumulation of drugs. Both types of NPs were then subjected to another coating with hybrid membranes (HM), derived from red blood cell cancer. The HM coating provided enhanced biocompatibility due to its biomimetic and homogeneous properties. Both systems were then administered sequentially to tumor-bearing mice and irradiated, presenting synergistic effect against tumor and metastasis [73]. PLGA also provides the possibility of encapsulating various PSs, belonging to completely different groups, such as Ce6, belonging to chlorins, and indocyanine green (ICG), belonging to the cyanin group. However, such a combination is known for mutually altering the activity due to fluorescence resonance energy transfer (FRET). In fact, ICG can perform PTT instead of PDT, and the factor that allows this combination to work properly is associated with ICG degradation during the photothermal process due to low photostability. A decrease in ICG concentration allows for maintaining the photodynamic effect by Ce6, which can be utilized for combined PTT/PDT of tumors [74]. The combined PDT/PTT approach may also be achieved by incorporation of polydopamine moiety, as well as the use of iron(III) salt of tannic acid outside the Ce6-loaded PLGA shell. Both approaches may exhibit high activity in combined treatment due to good targeting properties and high either photothermal or photodynamic efficacy [75,76].

**Table 2:** Advantages and disadvantages of polymer-based NP.

Type of Nanoparticle	Advantages	Disadvantages
Cellulose-based	Excellent biocompatibility and biodegradability suitable for biomedical applications [61].	Moderate drug release efficiency compared to free PSs [62].
Chitosan-based	High functional versatility and responsiveness to tumor microenvironment stimuli [65,66,67].	Reduced effectiveness against certain Gram-negative bacteria or structurally resistant cells [64].
Fibroin-based	Exceptional biodegradability and low immunogenicity [69].	Structural sensitivity to environmental conditions affecting therapeutic consistency [68].
Polyethyleneimine-based (PEI)	Strong functional interactions facilitating effective drug loading [69].	Potential cytotoxicity requiring additional modifications to improve biocompatibility [71].
PLGA-based	High versatility in encapsulation of diverse therapeutic molecules [73,74].	Potential interference in combined PS-drug systems (e.g., energy transfer issues) [74].

## 4. Carbon-Based Nanocarriers

**Figure 5:**
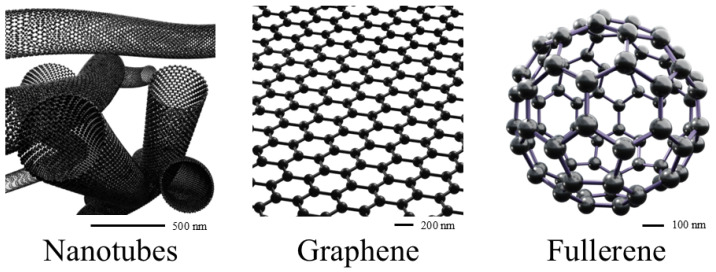
Representation of shapes of carbon-based nanocarriers. These images were created with the use of Blender 4.1.

Carbon-based nanocarriers (CBNCs) are also more than suitable to use in PDT. Similarly, to already mentioned groups they can help in increasing therapeutic efficiency, decreasing the effective dose of PS, and minimizing adverse effects. CBNCs can make use of different carbon forms like graphene, reduced graphene oxide, graphene oxide, graphene-based quantum dots, and carbon dots. Also, CBNCs vary widely in regard to their shapes: nanotubes, films, and particles (fullerenes, quantum dots, as shown in Figure 5) [77]. The structure of a CBNC has an influence on crucial properties of the complex, like deposition, binding potential, and stability. Small size enables cell membrane crossing, while bigger size and bigger surface area increase the binding potential and oxidative potential [77]. Another advantage of CBNCs is that they can be easily modified with other ligands in order to improve their solubility and specificity (Table 3). That said, some of the CBNCs (graphene oxide or graphene quantum dots) exhibit better aqueous solubility and biocompatibility [77].

In spite of their poor water solubility, carbon dot-based (CD) PSs can be easily obtained, e.g., by dissolving *Hypericum perforatum* dry extract in water, a mix of water and ethanol, or a mix of water, ethanol and polyethyleneimine (PEI) [78]. Another example of CBNCs successfully applied are graphene quantum-dot-loaded GQD with curcumin. Curcumin, the same as hypericin, is a naturally existing molecule with photosensitizing properties that can be used against bacteria. Curcumin’s water solubility and permeability are low; hence, it is more than suitable to couple it with GQD [79].

It is common to introduce different metal atoms to CBNCs to enhance their synergistic effect with PS. Fe or Zn atoms are commonly used as the addition to carbon-scaffold-forming metal–organic frameworks (MOFs). MOFs are a group of hybrid inorganic–organic crystals that consist of metal ions and organic ligands. Their microarchitecture and chemophysical properties can be changed via the selection of ligands and metal ions, which are used in the manufacturing process. This grants MOFs considerable versatility compared to other CBNCs. The addition of metal in MOFs enables them to serve as highly effective oxygen reduction catalysts, which synergistically improves PSs’ ability to generate ROS. The iron-based MOFs are able also to act as catalysts in Fenton-like reactions, causing degradation of H_2_O_2_ to HO^•^ and O_2_. This process is strongly desirable for PDT, helping to unlock PS’s full potential in the hypoxic tumor environment [80].

### 4.1. Chlorins and Graphene

Graphene oxide (GO) is an oxidized graphene derivative, utilized as a highly functionalized carrier for PDT PSs due to presence of various oxygen-based groups on its surface, including hydroxyl, carboxyl and epoxide groups. The functionalization of a graphene surface allows for achieving as high biocompatibility as possible, also incorporating various drugs and targeting them into tumor sites. Additionally, GO presents good photothermal properties, allowing for various combinations with PDT and other methods of treatment. Such a system based on GO may include a potent PSs, for example, Ce6, and some molecules providing good targeting properties. As tumor cells often do overexpress the folate receptors, modification with FA can enhance the delivery due to its high affinity to the receptor. Both Ce6 and FA can be linked with GO nanosheets through the short PEG chains utilizing amidation and esterification, respectively, while the PEG can be introduced by nucleophilic opening of the epoxide groups [81]. The PTT efficacy can be enhanced with proper modifications, such as incorporation of magnetite NPs. This can be achieved by triethylene glycol-assisted dispersion of iron(III) acetylacetonate. Then, a PS (Ce6) can be introduced into the obtained nanocomposite, and the whole system can be further modified with a specific aptamer to provide tumor-targeting properties. Additionally, to enhance the antitumor efficacy, the nanocomposite can be loaded with PTX to elevate the cytotoxic effect [82]. Broader functionalization of the GO surface for extended attachment capability can be achieved by conjugation with supramolecular moieties, allowing for a multimodal therapeutic approach. The supramolecular units can be delivered by conjugation of cucurbit[7]uril (CB[7]), which provides strong host–guest interactions with other molecules. Since the loading of aromatic compounds onto the GO can be performed by π–π stacking, the Ce6 and AQ4N were loaded this way, whereas the CB[7] units were utilized for encapsulation of a cytostatic drug, oxaliplatin (OX). The remaining CB[7] was then used for adamantane-substituted hyaluronic acid (HA) coating of the system to improve its stability. The combined multimodal approach exhibited good inhibition of tumor growth, indicating the advantage of such extended therapy [83].

### 4.2. Chlorins and Carbon Quantum Dots

Quantum dots (QDs) can be produced from diverse materials, giving various possibilities of conjugating PSs, as well as different properties useful for further treatment. Interestingly, CQDs obtained with some methods tend to exhibit the properties of its precursors, making it useful for maintaining the desired functions, such as specific targeting or photodynamic activity. Such specific CQDs were produced with the plasma electrochemical method using helium plasma created between tungsten steel tube delivering the gas, and the liquid medium with platinum electrode immersed in it. In this system, the tube and plasma of flowing helium acted as an anode, while the Pt electrode served as a cathode. The liquid medium was in fact the solution of two compounds desired to create CQDs: FA and Ce6. Such a combination provided good folate–receptor affinity and sufficient absorption of red light. While the dark toxicity was negligible, the PDT resulted in a significant reduction in tumor cell viability. Such effects indicate that the plasma electrochemical method may be a versatile approach in the synthesis of highly functionalized CQDs [84]. Another material suitable for the creation of QDs is black phosphorus (BP), used, for example, as a PPT agent in combining PTT/PDT with Ce6. The BPQDs were loaded into the lignin-based nanocarrier that was modified with Ce6 as PS and triphenylphosphine (PPh_3_) to ensure good mitochondria targeting. The system utilized the vulnerability of lignin to singlet oxygen and therefore provided the photo-induced opening of the carrier. The combined PTT/PDT approach of the developed system resulted in significantly better effects than both approaches alone, either greatly reducing the tumor cell viability or completely inhibiting tumor growth [85]. A similar approach utilized silver(I) sulfide as a photothermal agent along with Ce6, although their effects were additionally enhanced by CAT and OX. This strategy took advantage of pH-dependent electrostatic self-assembly of the precursors on the CAT surface and its TME-triggered disassembly, as the systems exhibited limited stability in lowered pH. This allowed for triggered release of drugs and their combined action, resulting in a high reduction in cell viability, especially in a hypoxic environment, and complete inhibition of tumor growth [86]. QDs can also serve as an efficient energy donor for PS via FRET for an enhancement in its efficiency. Such a system utilized two-layered QDs in which the core was composed of cadmium and selenium, and the shell was made of zinc(II) sulfide. This allowed for conjugation of modified PEG moieties, providing the binding of Ce6 and improved stability, depending on the introduced substituents. The conjugate was also modified with a membrane-targeting peptide (JB858), using its non-covalent affinity to the ZnS shell. Additionally, the hydrophilic QD improved the aqueous stability and solubility of the Ce6. The cell viability assay proved that QD-Ce6-JB858 had the highest cytotoxicity when irradiated both with 488 nm laser and 633 nm in comparison to QD-JB858 and Ce6 alone. What is interesting is that although Ce6 has minimal absorption in the 488 nm region, the effective engagement of the FRET system can still provide a photodynamic effect. To conclude, better targeting and the FRET PDT enhancement of such a system resulted in an improvement in Ce6 activity toward tumor cells [87].

**Table 3:** Advantages and disadvantages of carbon-based NP.

Type of Nanoparticle	Advantages	Disadvantages
Graphene Oxide (GO)	Excellent biocompatibility and functionalization potential (e.g., folic acid targeting) [81].	Stability issues due to possible aggregation or non-specific interactions in biological media [83].
Graphene Quantum Dots (GQDs)	Improved solubility and efficient loading of poorly soluble natural PS (e.g., curcumin) [79].	Complex preparation methods and potential variability in physicochemical properties [79].
Metal–Organic Frameworks (MOFs)	Enhanced catalytic ability to produce ROS (e.g., iron-based Fenton-like reactions) [80].	Possible heavy metal-associated cytotoxicity, necessitating careful biocompatibility evaluation [80].
Carbon Quantum Dots (CQDs)	Ability to incorporate multiple functions, including targeted drug delivery and ROS generation [84].	Potentially inconsistent photodynamic efficacy depending on preparation methods and precursor selection [84].
Black Phosphorus Quantum Dots (BPQDs)	High photothermal conversion efficiency for combined PTT/PDT applications [85].	Instability and rapid degradation under physiological conditions [85].
Metal-containing Quantum Dots (CdSe/ZnS)	Efficient energy transfer (FRET) for enhanced PS activation [87].	Concerns regarding heavy-metal toxicity and long-term biocompatibility [87].

## 5. Nanospheres

Nanospheres constitute a good platform for PDT, being prone to various bulky modifications in order to improve their properties and activity (Table 4). As an example, copper(II) sulfide spheres were surface-modified with hexachloroplatinic acid followed by reduction to free Pt (Figure 4). Then, the surface was modified with PEG chains and loaded with Ce6. The Pt on the surface presented CAT-like action, increasing the oxygen content due to intratumoral H_2_O_2_ decomposition, and therefore improved the effect of Ce6-mediated PDT. In addition, the CuS core of the nanosphere was prone to the photothermal effect, allowing for synergistic PTT/PDT action toward tumors [88]. The mesoporous silica nanospheres (MSN) are also a versatile approach, allowing for the dispersion of metals that have enzyme-like properties to enhance PDT efficiency. Incorporation of dual metallic sites composed of manganese and gadolinium on the MSNs surface, due to creating the electron–hole pairs, can provide CAT-like activity, elevating the oxygen content, but also a peroxidase and oxidase-like performance, resulting in additional generation of radicals. Moreover, such a system is capable of catalyzing the conversion of superoxide radicals to singlet oxygen, increasing the antitumor efficacy and completely inhibiting its growth [89]. The MSN can also constitute an outer shell of the specific metal-based core, providing the possibility of effective Ce6 loading. L. Palanikumar et al. achieved a core composed of lanthanides (ytterbium, gadolinium, and erbium) and their combinations with bismuth selenide (NaYF_4_:Yb/Er/Gd,Bi_2_Se_3_), forming a photothermal agent. The lanthanide core was designed for the successful conversion of NIR radiation into visible light for activating the PS, whereas the Bi_2_Se_3_ was supposed to convert the NIR radiation directly into heat for PTT performance. The system with loaded Ce6 was additionally coated with lipids to retain enclosed Ce6 and reduce the adsorption of serum proteins, and recognition by the macrophages, forming Ce6-loaded lipid-coated, upconversion mesoporous silica nanospheres (Ce6-LUMSN). Moreover, it was linked with a pH-responsive rational membrane peptide to further improve targeting and internalization properties. The proposed system was tested for its photothermal properties, resulting in the rise in the buffer temperature up to 55.5 °C for the 200 μg/mL (concentration of Ce6-LUMSN) irradiated with 1.5 W/cm^2^ NIR laser for 5 min. Ce6 cargo release was also characterized; the sequence with three NIR laser irradiations (each lasting 10 min) achieved the highest release rate, of almost 100%, throughout 24 h. The Ce6-LUMSN functionalized with ATRAM (Ce6-ALUMSN) was tested in vivo in 4T1 (human breast cancer) tumor-bearing mice, exhibiting favorable pharmacokinetics, achieving the highest Ce6 tumor cell concentration, compared to Ce6-LUMSN or Ce6 alone. This also resulted in a significantly higher survival rate of mice in the Ce6-ALUMSN group and excellent tumor volume reduction over 30 days past the intervention [90].

## 6. In Vivo Research Progress

Although the first human trials with PDT were conducted more than a century ago, at the moment, there is only one photosensitizing drug (VISUDYNE^®^) registered in the form of a combination of PS and liposomes [2]. According to site the www.clinicaltrials.gov, 72 registered clinical studies used VISUDYNE^®^ in their protocols. That being said, there is still a huge gap in clinical knowledge and in vivo trials when it comes to the combination of chlorin-based PSs with nanotechnology-based enhancers.

### 6.1. Temoporfin

A study on PDT of feline cutaneous squamous cell carcinoma was conducted by Buchholz et al. The authors tested a liposomal formulation of mTHPC on a group of 18 animals with low-stage tumors (between T1a and T2b). The animals received a liposomal formulation of mTHPC with a dose equal to 0.15 mg/kg of body weight, which was administered via the cephalic or femoral vein over 10 min. The PDT was performed 16 h or 6 h after injection, with a 652 nm diode laser coupled with a quartz fiber with a microlens as the light source. The light dose was 10 J/cm^2^ over 200 s, with the laser set to a power density of 0.05 W/cm^2^. Prior to irradiation, the animals were premedicated and anesthetized. The clinical image of the animals was assessed before and after treatment, revealing no changes in serum biochemistry, nor the occurrence of adverse reactions. Only three animals (15%) suffered from acute local adverse effects, with the rest experiencing mild edema and erythema that lasted up to 4 days after the treatment. The initial response rate was 100%, and only four animals (20%) recurred, with a median time of 172.0 (±87.1) days. The results obtained by Buchholz et al. for the overall 1-year local control rate were 75% [91]. Millard et al. compared and evaluated the pharmacokinetics and PDT effects of the commercial liposomal formulation of mTHPC (Foslip^®^), and mTHPC-Extracellular Vesicles (mTHPC-EV) on HT-29 (human colon adenocarcinoma) xenograft mice. Moreover, the authors presented a method of obtaining mTHPC-EV with high loading capacity by using human umbilical vascular endothelial cells. The change in mTHPC tumor-bearing mice plasma concentration over time after injection revealed a significant difference between Foslip^®^ and mTHPC-EV. After intravenous injection of 0.3 mg/kg, the highest plasma concentration of free mTHPC for Foslip^®^ was at the 30 min time mark, reaching an average of 1.5 ng/mL and rapidly decreasing to a concentration of less than 0.2 ng/mL at the 6 h time mark. In contrast, the same dose of mTHPC-EV administered in the same way seems to achieve prolonged release, reaching the peak concentration of 0.8 ng/mL as late as around 5–7 h after the injection. Moreover, whole-body fluorescent imaging of animals revealed that mTHPC-EV possesses a more desirable biodistribution, with a higher accumulation rate in tumor tissue than Foslip^®^ measured at the 6 h mark. The authors defined remission as the absence of tumor regrowth at 90 days after treatment, giving the best performance (33%, 3/9) to animals cured by mTHPC-EV with PDT performed at 24 h after administration. In comparison, the best-performing Foslip^®^ group was the one with PDT performed at 6 h after administration, with (27%, 2/7) animals cured [92].

### 6.2. Chlorin e6

Promising in vivo results are presented by Huang et al., who combined Ce6 with self-assembling pH/ROS dual-responsive NPs. The authors developed a copolymer based on pH-sensitive PBAE (poly(β-amino ester) and an ROS-sensitive thioketal (TK) linker, forming mPEG-TK-PBAE. The authors decided to test mPEG-TK-PBAE, Ce6 and triptolide (TPL) as a combination (TPL/Ce6 NPs) of chemo-photodynamic therapy in H22 (mouse hepatoma cancer) xenograft nude mice. TPL is a diterpenoid found in Tripterygium wilfordii (medicinal herb), and is known for anti-tumor activity against a range of hepatocellular carcinoma cell lines [93]. The in vivo distribution of TPL/Ce6 NPs was measured in tumor-bearing mice after intravenous injection with the help of fluorescent imaging. The TPL/Ce6 NPs group exhibited fluorescence predominantly in tumor site, as early as 2 h post administration, which persisted until the 24 h post administration. In comparison, the free Ce6 group demonstrated less selective distribution toward tumorous tissue; however, fluorescence was present after 24 h post injection mainly in the liver region, with minimal intensity around tumor site. The animals were treated through a 14-day period and were administered the corresponding drug or control solution every other day; the TPL/Ce6 NPs animals were irradiated at 3 h and 12 h post injection, with a 650 nm 0.63 W/cm2 laser for 5 min. After the study, the TPL/Ce6 NPs group exhibited the lowest tumor weights and volumes, compared to other groups, being the only group to completely inhibit and decrease the size of the malignancy [93]. Chemo-photodynamic therapy with NPs was also used by Ding et al., who tested dextran-based micelles loaded with Ce6, and with modified PTX (ROS-activable thioketal prodrug), forming (PCL-NPs) in an orthotopic 4T1 (murine stage IV breast cancer) BALB/c female mice model. Rather than depending on the Ce6 photodynamic activity, they used it as an activator for the ROS-sensitive PTX prodrug via internally induced chemiluminescence. The approach included the administration of luminol (3-aminophthalhydrazide), which can act as an in situ chemiluminescent agent in the presence of a high concentration of H_2_O_2_. This can cause ^1^O_2_ generation by Ce6 that not only damages intracellular structures but can also activate the PTX prodrug. The results of fluorescent imaging show that after intravenous injection, PCL-NPs could effectively accumulate in tumors. Moreover, the PCL-NPs outperformed the in vivo monotherapy control (PTX) by more effectively inhibiting tumor growth in size and weight. Also, in the pathomorphological analysis, the PCL-NPs group exhibited the highest apoptosis and lowest proliferation levels in the cancerous tissues, implicating that it possesses stronger antitumor activity [94]. Artsemyeva and Tzerkovsky (2023) [95] conducted a safety and efficacy study in humans, evaluating chlorin-based photosensitizer “Photolon” (Ce6 trisodium salt complex with polyvinylpyrrolidone) [95]. They tested the PDT protocol in 172 patients with stage 1 basal cell carcinoma (BCC). Intravenous administration of Photolon (2.0–2.5 mg/kg) was followed by tumor irradiation (660 ± 5 nm or 665 ± 5 nm; 50–250 J/cm^2^) after 2.5–3 h. The treatment exhibited exceptional tolerability, with only 5.8% of patients experiencing transient grade I-II skin phototoxicity (hyperemia, edema) and no severe systemic adverse events. Morphological assessment at 3 months post-PDT revealed complete tumor regression in 93.0% of cases and partial regression in 4.7%, yielding a 97.7% objective response rate. The long-term recurrence rates remained low (3.1% at 1–2 years; 6.9% at 5 years) [95]. Cui et al. published a paper on developing a PEI-capped upconversion system with manganese nanoparticles (Ce6-MnNPs; ~55 nm) to enhance chlorin e6 delivery for NIR-PDT against oral squamous carcinoma (HSC-3 cells). The Ce6-MnNPs complex mitigated Ce6’s hydrophobicity and optimized cellular internalization; moreover, it enabled efficient energy transfer under 980 nm irradiation (0.5 W, 20 s). NIR-PDT significantly suppressed HSC-3 proliferation and induced apoptosis via mitochondrial pathway dysregulation, which was proven by reduced membrane potential, elevated Bax/Bcl-2 mRNA ratio, and nuclear p53 accumulation. Despite marked cytotoxicity and ROS generation, the authors noted limitations in clinical scalability due to nanoparticle aggregation tendencies and high synthesis costs [96]. Another study by Tzerkovsky et al. also investigated chlorin-based photosensitizer “Photolon” as a radiosensitizer for radiodynamic therapy (RDT). The study included rats bearing Pliss lymphosarcoma (PLS) tumors. Combining Photolon with γ-radiation (^192^Ir source; 2–6 Gy single doses) significantly potentiated antitumor efficacy versus radiation alone. The 6 Gy co-treatment cohort achieved maximal tumor growth inhibition (TGI = 57.61%) and 40% complete regressions at 60 days. The radiodynamic activity was statistically confirmed at 4 Gy. Mechanistically, PS-mediated ROS amplification under ionizing radiation impaired tumor cell repair mechanisms. This study highlights chlorins’ utility in RDT but underscores dose-dependent efficacy thresholds, with no benefit observed at 2 Gy [97].

## 7. Summary

Chlorins are the most promising group of photosensitizers for photodynamic treatment (PDT, PACT). They reveal appropriate properties, like reasonable absorption in the so-called “therapeutic window” and a relatively high ability to form reactive oxygen species. This class of compounds has been successfully introduced to the clinics. It should be mentioned here that chlorin e6 is used in the photodynamic treatment of gliomas and lung cancer, and Foscan^®^ is used against head and neck, pancreatic, and prostate cancers. Nevertheless, chlorins are prone to decomposition, and based on their structure, they can hardly be soluble in the water environment. Therefore, there are many attempts to prepare new formulations composed of lipids, polymers, nanoparticles and many other carriers. The most explored are liposomes presenting a versatile nanoplatform for PDT (strong encapsulation capability, ability to co-delivering drugs). However, they may exhibit limited physicochemical stability and relatively reduced targeting specificity. Thus, the careful optimization of liposomal formulations remains essential to fully exploit their therapeutic potential in PDT. The second popular cargo searched for PDT application is micelles, enabling efficient encapsulation of PS alongside cytostatic drugs and allowing for precise control over drug release in response to TME stimuli such as ROS or low pH. Despite their notable versatility and improved responsiveness, micellar formulations can be limited by insufficient ROS generation in vivo or complex stability challenges, potentially reducing therapeutic predictability. This is not without significance, however, as PSs carriers are also polymer-based NPs, carbon-based, nanospheres and many others.

Throughout this paper, various nanotechnology-based enhancing approaches were mentioned, each of them offering specific advantages but also drawbacks due to their characteristics. To conclude, the current research seems to mainly focus on improving solubilization and cell membrane permeability. However, carriers that enable combining the PS and another drug into dual chemo-photodynamic systems are leading in terms of high activity results, both in in vitro and in in vivo models.

## Figures and Tables

**Table 4:** Advantages and disadvantages of nanospheres.

Type of Nanoparticle	Advantages	Disadvantages
Copper(II) sulfide-based nanospheres (CuS-Pt)	Synergistic photothermal and photodynamic action enhanced by CAT-like platinum activity [88].	Potential stability issues due to complex surface modifications [88].
Mesoporous silica-based nanospheres (Mn-Gd MSNs)	Multifunctional catalytic activity (CAT-, oxidase-, peroxidase-like) enhancing ROS generation and PDT efficiency [89].	Complex preparation procedure and potential concerns regarding biocompatibility of multiple metals [89].
Lanthanide-doped MSN-based nanospheres (NaYF_4_:Er,Yb,Gd@Bi_2_Se_3_)	Efficient NIR-triggered simultaneous PDT/PTT with controlled Ce6 release [90].	Risk of toxicity and stability issues due to lanthanide and heavy metal core components [90].

## Abbreviations

The following abbreviations are used in this manuscript:

PDT	photodynamic therapy
PS	photosensitizer
PLGA	Poly Lactic-co-Glycolic Acid
ROS	reactive oxygen species
Ce6	chlorin e6
mTHPC	temoporfin
NP	nanoparticles
aPDT	Antimicrobial Photodynamic Therapy
TGF-β1	Transforming Growth Factor beta 1
PACT	Photodynamic Antimicrobial Chemotherapy
MB	methylene blue
PTX	paclitaxel
CAT	catalase
3BP	3-bromopyruvate
TME	tumor microenvironment
AQ4N	banoxantrone dihydrochloride
MIC	Minimal Inhibitory Concentration
MBC	Minimal Bactericidal Concentration
NIR	near infrared light
MRSA	methicillin-resistant Staphylococcus aureus
UCON	ultra-small copper oxide
PEG	polyethylene glycol
SRF	sorafenib
CPT	camptothecin
PFCEs	perfluorinated crown ethers
GSH	glutathione
PBC	polymer-based complexes
PPDO	Poly(p-dioxanone)
PHB	Poly(hydroxybutyrate)
PHBV	Poly(hydroxybutyrate-co-hydroxyvalerate)
PBS	Poly(butylene succinate)
TNBC	triple-negative breast cancer
CMC	carboxymethyl cellulose
PC	pectin
SA	sodium alginate
Cp6	chlorin p6
HPCS	hydroxypropyl chitosan
SF	silk fibroin
DA	dopamine
CDI	CMC-polydopamine-NaI
PTT	photothermal therapy
PEI	polyethylenimine
GQDs	graphene quantum dots
HM	hybrid membranes
ICG	indocyanine green
FRET	fluorescence resonance energy transfer
CBNC	carbon-based nanocarriers
CD	carbon-dot-based
MOFs	metal–organic frameworks
GO	graphene oxide
CB[7]	cucurbit[7]uril
OX	oxaliplatin
HA	hyaluronic acid
QDs	quantum dots
BP	black phosphorus
EV	extracellular vesicles
TPL	triptolide
MSN	mesoporous silica nanospheres
PCL-NPs	biocompatible dextran based—multi-component nanomedicine
BALB/c	albino, laboratory-bred strain of the house mouse
DNA	deoxyribonucleic acid
4T1	human breast cancer cell line
WHO	World Health Organization
FDA	Food and Drug Administration
BCC	basal cell carcinoma
RDT	radiodynamic therapy
PLS	Pliss lymphosarcoma
